# Active Pressure Ripple Reduction of a Self-Supplied Variable Displacement Pump with Notch Least Mean Square Filter

**DOI:** 10.3390/mi12080932

**Published:** 2021-08-05

**Authors:** Xiaochen Huang, Bing Xu, Weidi Huang, Haogong Xu, Fei Lyu, Qi Su

**Affiliations:** The State Key Laboratory of Fluid Power and Mechatronic Systems, Zhejiang University, Hangzhou 310027, China; xiaochenh@zju.edu.cn (X.H.); bxu@zju.edu.cn (B.X.); x635709499@zju.edu.cn (H.X.); feilv@zju.edu.cn (F.L.); suqi@zju.edu.cn (Q.S.)

**Keywords:** variable displacement pump, pressure ripple, active control, least mean square (LMS) filter

## Abstract

As the power sources in hydraulic systems, variable displacement axial piston pumps generate flow fluctuation. Unfortunately, it results in pressure pulsation which excites the system vibration and emitted noise. The majority of studies try to eliminate the pulsation via a passive technique and the active control methodology has not been discussed in detail. In this research, the feasibility of reducing the pressure ripple by properly controlling the proportional valve has been investigated, which also supports the miniaturization of the active control system. A mathematical model of the self-supplied variable displacement pump including the control system has been developed. The filtered-X least mean square algorithm with time-delay compensation is utilized to calculate the active control signal. Simulation results show the effectiveness of the active control technique. The effect of the active control signal on the flow rate from different chambers of the pump has been analyzed. It demonstrates that the variation of the pressure pulsation should be ascribed to the comprehensive reaction of different flow rates. The major reason is that the flow of the actuator piston neutralizes the peak value of the flow ripple, generated by the nine pistons.

## 1. Introduction

Hydraulic systems usually utilize the variable displacement axial piston pumps as the power sources owing to their high power density, high efficiency, and controllability [1]. As volumetric machines, axial piston pumps discharge an oscillating flow rate, which generates the pressure ripple in hydraulic circuits [2]. It leads to vibration and noise in the pump component and the pipelines, degrading the reliability and durability [3,4]. In aircraft, the failures of hydraulic systems have been reported to be related to the vibration of pipes [5]. The sharp noise of the axial piston pumps in submarines would increase the possibility of being captured by sonar [6].

As the pressure ripple is a huge drawback in hydraulic systems, special attention has been paid to the techniques to reduce the amplitude of the pressure ripple. Actually, the pressure ripple stems from the pressure and flow fluctuation in the piston chamber, whilst the valve plate is the key component affecting the variation of the flow and pressure in the piston chamber [7]. The structure parameters have proved to influence the flow ripple and vibration of the piston pumps [8,9]. Therefore, in axial piston pumps, there is a large volume of published studies describing the geometric optimization of the valve plate—the transition regions between the inlet and outlet ports, to be exact. Different types of relief grooves have been studied to realize a smooth pressure transition in the piston chamber with optimal algorithms [10,11]. For example, damping hole, triangular groove, rectangular groove, and a combination of them. Special attention has been paid to the swash plate. A small, inclined angle of the swash plate called the cross-angle was introduced to reduce the flow ripple and emitted noise by Johansson et al. [12] and Liselott [13]. However, as a passive technique, the optimal design is hard to satisfy the demands of variable displacement pumps, which often operate at different working conditions. 

Notice that the methods described above are devoted to the source of the pressure ripple. Naturally, efforts can also be made in the path of pressure transmission. Though the passive pulsation dampers are the possible solutions [14,15,16], as the passive technique, there are still some difficulties under varying operation condition. Luckily, the active control of fluid pressure ripple has been explored by researchers using diverse types of actuators in hydraulic pipe system. Guan et al. [17] summarized these active control techniques of pressure pulsation and designed a piezoelectric direct-drive slide valve as the active vibration absorber. Zhai et al. [18] utilized the active constrained layer damping treatment to achieve the vibration control. Pan et al. [19] applied adaptive notch filters for pressure pulsation attention in a switched inertance hydraulic system, and a hybrid control system was also tried to reduce fluid-borne noise by integrating an active attenuator with passive flexible hoses [20]. Although the pressure pulsation reduces, additional control valves or actuators are necessary, which increases the complexity of the system.

Inspired by these, a few researchers investigate the active control methods utilizing the control valve of the variable displacement pump itself. Ohuchi et al. [21] proposed an active noise control method by controlling the swash plate vibration using the servo valve. The adjustment of the sinusoidal signal depended on the manual adjustment. On this basis, Ivantysynova et al. [22,23] achieved the adjustable reduction of the swash plate vibration by utilizing an adaptive least mean squares (LMS) filter to calculate the weight of the reference signal. Additionally, the noise reduction was verified experimentally at different operation conditions [24]. Recently, Casoli et al. [25] theoretically studied the possibility of active pressure ripple control by actively moving the swash plate. The control strategies were to decrease the swash plate angle when the instantaneous pressure is higher than the mean value and vice versa. Although the active vibration and noise control by actively controlling the swash plate has proven to be effective, the suppression principle has not been seriously revealed. To the best of our knowledge, the effect of the variable mechanism on pressure ripple has been overlooked, or the research object is the variable displacement pump with actuating system supplied externally. Extensive research has shown that for a variable displacement pump with the control flow supplied internally, the swash plate oscillation was considered to be influenced by the variable control mechanism [26,27].

Therefore, the aim of this essay is to give a comprehensive description of the active pressure control (APC) for a self-supplied variable displacement axial piston pump. To accomplish the APC, no additional mechanism is needed except its control valve. The influence of the active controller on pressure ripple is analyzed to figure out the reason for the pressure ripple reduction. The structure of this paper is as follows: Section 2 describes the self-supplied variable displacement axial piston pump and establishes a dynamic model of the variable displacement axial piston pump. The dynamics of the control mechanism and pumping piston is taken into consideration. Section 3 introduces the control strategy of APC with LMS algorithm to calculate the additional active control signal. Results of the pressure ripple have been shown with single-frequency and multi-frequency component APC. Section 4 presents a detailed analysis of the proposed method. By analyzing the different flow rates to the discharge chamber, the reason for the pressure pulsation reduction is revealed. Finally, Section 5 provides some comments and outlook for APC.

## 2. Mathematical Model

### 2.1. Description of the Physical System

**Figure 1:**
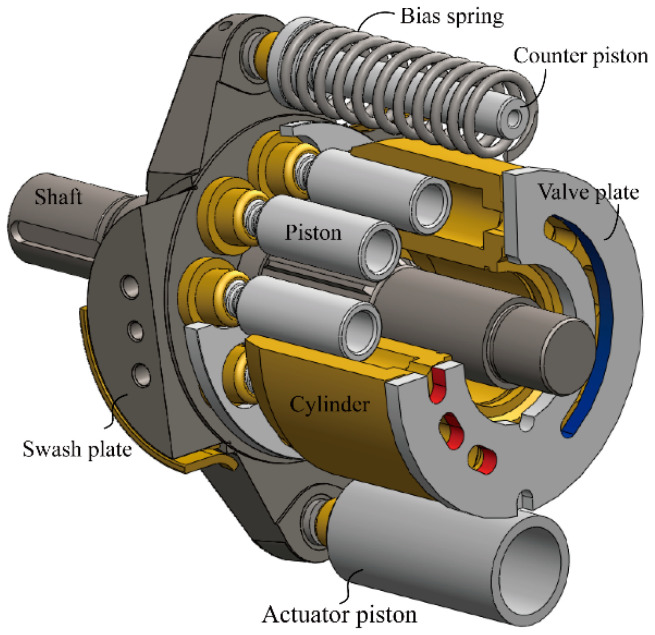
Schematic of the variable displacement pump.

As shown in Figure 1, the variable displacement axial piston pump is chiefly comprised of the rotating groups and the swash plate control mechanism. The rotating groups, cylinder and pistons included, rotate with shaft driven by the prime mover. Owing to the inclination of the swash plate, the piston executes the reciprocating motion inside the cylinder block. The valve plate assigns the piston flow rate to the inlet port and outlet port. The piston number connecting to the discharge port is finite, which generates a non-uniform flow rate that flows into the load system. As a result, the inevitable flow fluctuation is generated, and also the pressure in the piston chamber fluctuates. The pump shell vibrates after the oscillating forces are transmitted to the shell. 

**Figure 2:**
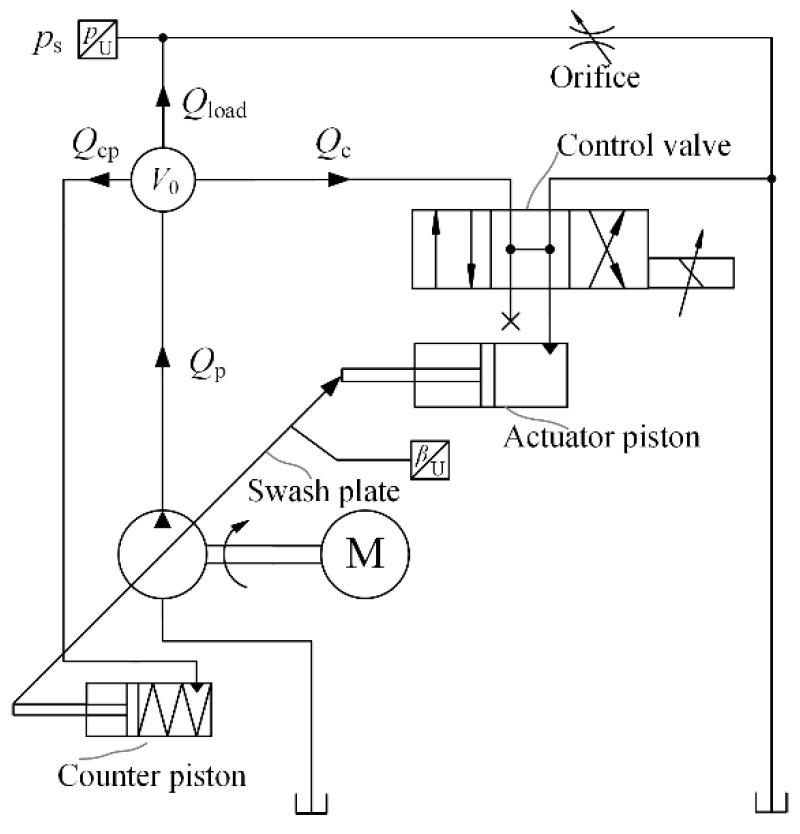
The control circuit of the self-supplied variable displacement pump.

As a key component of forming the pump displacement, the swash plate is regulated by the control mechanism. Here, the actuator piston, counter piston, and bias spring are utilized. Figure 2 gives a presentation of the pump control principle. The counter piston is directly connected to the discharge chamber. The hydraulic force and spring force drive the swash plate to increase the inclined angle. At the other side of the swash plate, the hydraulic force of the actuator piston plays an opposite role in changing the angle, and in order to adjust the control force, a proportional valve is utilized to control the pressure in the actuator piston chamber. The control flow stems from the discharge chamber of the pump itself, which is called a self-supplied variable displacement pump. In this research, the pressure control is realized by the PID controller.

### 2.2. Axial Piston Pump

As mentioned above, the pressure ripple originates from the flow fluctuation of pistons. The relationship between the pressure in the piston chamber and the flow rate of each piston is required to be defined by applying the continuity equation in integral form as follows [7]:

(1) $$\frac{dp_{i}}{dt}=\frac{K_{e}}{V_{p,i}}(q_{\mathrm{in}}-q_{\mathrm{out}}-q_{\mathrm{lk}}-\frac{dV_{p,i}}{dt})$$

where *dp_i_*/*dt* is the variation rate of pressure in piston chamber, subscript *i* represents the *i*-th piston, *K*_e_ is the fluid bulk modulus, *V*_p,*i*_ is the changing volume of the *i*-th piston chamber, *q*_lk_ is the sum of leakages through three different friction pairs, *q*_in,*i*_ and *q*_out,*i*_ are the flow rates between the piston chamber and the pump port. It can be described as:

(2) $$q_{\mathrm{in},i}=CA_{\mathrm{LP}}\sqrt{\frac{2\left|P_{\mathrm{in}}-p_{i}\right|}{\rho }}\cdot sgn(P_{\mathrm{in}}-p_{i})$$

(3) $$q_{\mathrm{out},i}=CA_{\mathrm{HP}}\sqrt{\frac{2\left|P_{\mathrm{out}}-p_{i}\right|}{\rho }}\cdot sgn(p_{i}-P_{\mathrm{out}})$$

where *C* is the flow coefficient, *A*_LP_ and *A*_HP_ are the opening areas between the piston chamber and pump port, *P*_in_ and *P*_out_ are pressures in the inlet and outlet chambers, *ρ* is the fluid density. The fluid properties such as fluid density and bulk modulus is taken into consideration according to [7].

By summing up the flow rates from nine pistons, the discharge flow rates of the piston pump can be obtained by:

(4) $$Q_{p}=\sum_{i=1}^{N}q_{\mathrm{out},i}$$

where *N* is the piston number. 

The gradient of the chamber volume is the cause of the discharge flow. It relies on the movement of the piston *z_i_*, which can be described as [4]:

(5) $$z_{i}=R\sin\varphi_{i}\tan\beta$$

where *R* is the piston pitch radius, *φ_i_* is the rotation angle of the *i*-th piston, *β* is the inclined angle of the swash plate. 

The motion, including the rotation of the piston and the swing of the swash plate, brings about the variation of the displacement. Thus, the velocity of the piston can be figured out, as follows:

(6) $${\dot{z}}_{i}=R\omega \cos\varphi_{i}\tan\beta +\frac{R\sin\varphi_{i}}{\cos^{2}\beta }\frac{d\beta }{dt}$$

### 2.3. Swash Plate Control System

**Figure 3:**
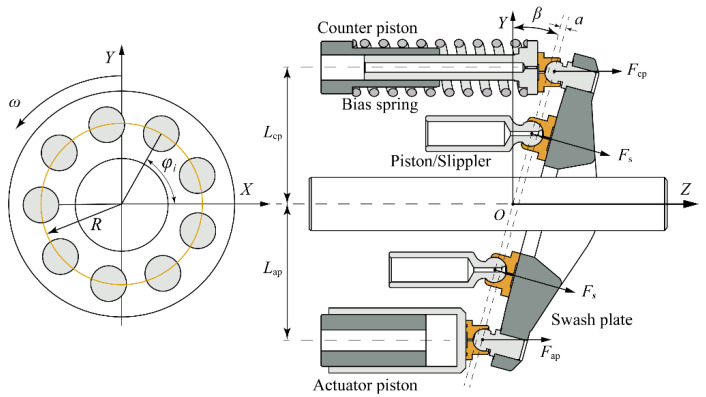
Schematic of swash plate control system.

In a variable displacement pump, the discharge flow rate varies with the angle of the swash plate. As shown in Figure 3, the swash plate angle of the variable displacement pump is decided by the control moments, which are generated by the pumping piston and control mechanism. The dynamics of the swash plate can be expressed as [20]:

(7) $$J_{\mathrm{sw}}\frac{d^{2}\beta }{dt^{2}}+c_{\mathrm{sw}}\frac{d\beta }{dt}=M_{p}+M_{\mathrm{ap}}+M_{\mathrm{cp}}$$

where *J*_sw_ is the rotational inertia of the swash plate around X-axis, *c*_sw_ is the damping coefficient of the swash plate, *M*_p_*, M*_ap_, and *M*_cp_ are the moments generated by the nine pistons, an actuator piston, and a counter piston, respectively.

The support force acting on the piston should be calculated for evaluating the moment. Thus, the analysis of the piston dynamics is utilized to calculate the support force which can be expressed as:

(8) $$p_{i}\frac{\pi }{4}d^{2}-f_{i}-F_{\mathrm{si}}\cos\beta =m_{i}{\ddot{z}}_{i}$$

where *d* is the diameter of the piston, *f_i_* is the friction force, *F*_si_ is the support force of swash plate, *m_i_* is the mass of the piston and slipper. 

The moment *M*_p_ which mainly generated by pumping pistons and can be described as follow [20]:

(9) $$M_{p}=\sum_{i=1}^{N}\frac{R\sin\varphi_{i}}{\cos\beta }F_{\mathrm{si}}$$

In the same way, the motion of the actuator piston must be modeled to get the actuator piston force. The motion equation for the actuator piston can be written as:

(10) $$m_{\mathrm{ap}}{\ddot{x}}_{\mathrm{ap}}=p_{c}A_{\mathrm{ap}}-f_{\mathrm{ap}}-F_{\mathrm{ap}}$$

where *m*_ap_ is the actuator piston mass, *x*_ap_ is the displacement of the actuator piston, *f*_ap_ is the friction force, *p*_c_ is the pressure in actuator piston chamber, *F*_ap_ is the force acting on the swash plate by the actuator piston.

The moment acting on the swash plate due to the actuator piston can be described as:

(11) $$M_{\mathrm{ap}}=F_{\mathrm{ap}}\left(L_{\mathrm{ap}}\cos\beta +a\sin\beta \right)$$

where *L*_ap_ is the distance between the shaft and actuator piston, *a* is the eccentric distance of the ball joints.

Besides, the continuity equation is utilized to deduce the pressure in the actuator piston chamber *p*_c_. It can be written as:

(12) $$\frac{V_{\mathrm{ap}}}{K_{e}}\frac{dp_{c}}{dt}=Q_{\mathrm{ap}}-A_{\mathrm{ap}}{\dot{x}}_{\mathrm{ap}}-Q_{\mathrm{apl}}$$

where *V*_ap_ is the volume of the actuator piston chamber, *Q*_ap_ is the flow rate between the control valve and the actuator piston, *A*_ap_ is the area of the actuator piston, and *Q*_apl_ is the leakage.

The relationship between the actuator piston displacement and the swash plate angle:

(13) $$x_{\mathrm{ap}}=a\cos\beta -L_{\mathrm{ap}}\sin\beta -a$$

Similarly, to calculate the force acting on the swash plate by the actuator piston *F*_cp_, the kinematics and dynamics of the counter piston are expressed as:

(14) $$m_{\mathrm{cp}}{\ddot{x}}_{\mathrm{cp}}=p_{\mathrm{cp}}A_{\mathrm{cp}}-f_{\mathrm{cp}}+F_{\mathrm{sp}}-F_{\mathrm{cp}}$$

(15) $$x_{\mathrm{cp}}=a\cos\beta +L_{\mathrm{cp}}\sin\beta -a$$

(16) $$\frac{V_{\mathrm{cp}}}{K_{e}}\frac{dp_{\mathrm{cp}}}{dt}=Q_{\mathrm{cp}}-A_{\mathrm{cp}}{\dot{x}}_{\mathrm{cp}}-Q_{\mathrm{cpl}}$$

where *m*_cp_ is the counter piston mass, *x*_cp_ is the counter piston displacement, *p*_cp_ is the pressure in the counter piston chamber which equals to the discharge pressure *p*_s_, *A*_cp_ is the area of the counter piston, *f*_cp_ is the friction force, *F*_sp_ is the force of the bias spring, *F*_cp_ is the force acting on the swash plate by the counter piston, and *L*_cp_ is the distance between the shaft and counter piston, *V*_cp_ is the volume of the counter piston chamber, *Q*_cp_ is the flow rate of the counter piston chamber, and *Q*_cpl_ is the leakage.

As a result, the moment caused by the counter piston is depicted by:

(17) $$M_{\mathrm{cp}}=F_{\mathrm{cp}}\left(L_{\mathrm{cp}}\cos\beta -a\sin\beta \right)$$

The dynamics of the proportional valve is simplified as a second-order model. It can be written as [22]:

(18) $$\frac{x_{v}}{u}=\frac{k_{v}\omega^{2}}{s^{2}+2\xi \omega +\omega^{2}}$$

where *x*_v_ is the displacement of the proportional valve, *u* is the control signal, *k*_v_ is the gain, *ζ* is the damping ratio, and *ω* is the natural frequency.

Then, the flow rate entering and leaving the actuator piston chamber via the proportional valve, *Q*_ap_ is conducted as:

(19) $$Q_{\mathrm{ap}}=\left\{ \begin{array}{l} k_{q}x_{v}\sqrt{\left(p_{s}-p_{c}\right)/\rho }\;x_{v}\geq 0\\ k_{q}x_{v}\sqrt{p_{c}/\rho }\;x_{v}<0 \end{array}\right.$$

where *k*_q_ is the flow coefficient of the valve.

### 2.4. Model Validation

**Figure 4:**
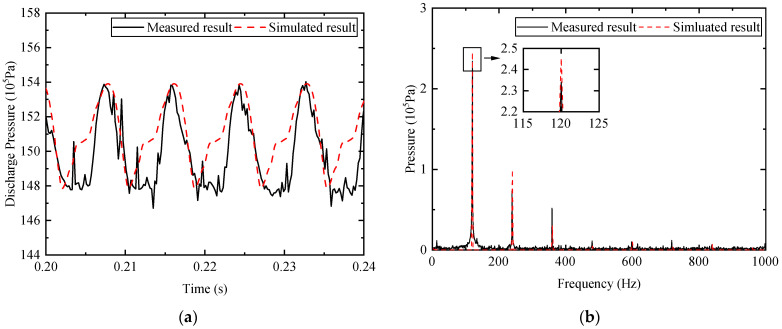
Comparison of the discharge pressure: (**a**) time domain; (**b**) frequency domain.

Before conducting the simulation research, experimental research using PID controller has been carried out to validate the model. A pressure transducer has a bandwidth of 5 kHz, which is located at the pump outlet for the measurement of the pressure ripple. By using a Labview program via NI 6218, experiment data is acquired. Figure 4 compares the discharge pressure under the working condition of 800 rpm rotational speed, 12° swash plate angle, and 15 MPa outlet pressure. Both measured and simulated pressure fluctuate around 14.8 to 15.4 MPa. From the graph of the fast Fourier transform (FFT) of the pressure ripple, both results show a peak value at the harmonic frequency, which is nine times frequency of rotational shaft. From the perspective of the amplitude, the first- to third-order frequency harmonics are the main components of the pressure ripple. Although the simulated pressure wave shape is not entirely consistent with measured result and neither is the peak amplitude, the simulation model is capable of investigating the methodology of the APC. The explanation for this might be that the pressure ripple is also concerned about the pipeline impedance and the position of the pressure transducer.

## 3. Active Pressure Ripple Control

### 3.1. Active Pressure Controller

**Figure 5:**
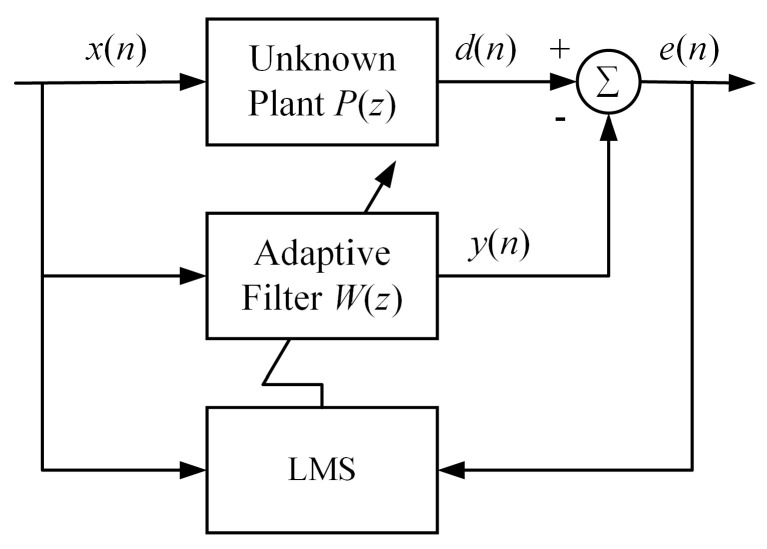
Schematic diagram of an active noise and vibration control system.

In the active noise and vibration control system, an adaptive filter is utilized as a canceller which is adjusted by the LMS algorithm. As it is shown in Figure 5, the desired signal *d*(*n*) takes off the filter output *y*(*n*) to get the residual error *e*(*n*). For *e*(*n*) decreasing to zero, the LMS algorithm is used to change the weight of the adaptive filter *W*(*z*). The LMS algorithm, which was originally proposed by Widrow and Hoff [28], is widely used in active noise and vibration control owing to its stability and simplicity.

The weight is updated as follows [28]:

(20) w(n+1)=w(n)+μx(n)e(n)

where the ***w***(*n*) is the weight vector of the adaptive filter, *μ* is the convergence coefficient, ***x***(*n*) is a reference input vector. 

The filter output signal is calculated by:

(21) $$y\left(n\right)=w^{T}\left(n\right)x\left(n\right)$$

Thus, the residual error is written as:

(22) e(n)=d(n)−y(n)

The reference input should be able to characterize the noise signal for a good performance of noise reduction. In the narrowband active noise control system, noise sources are usually periodic. In such circumstance, the reference input can be a sinusoidal signal with specified frequency and harmonics [29]. An adaptive notch filter can be achieved by using:

(23) $$x\left(n\right)={\left[A_{0}\sin\left(\omega_{0}n\right)\;A_{0}\cos\left(\omega_{0}n\right)\;A_{1}\sin\left(\omega_{1}n\right)\;A_{1}\cos\left(\omega_{1}n\right)\;\ldots \right]}^{T}$$

where *A*_0_, *A*_1_ are the amplitudes and *ω*_0_, *ω*_1_ are the frequencies. In an axial piston pump, the principal frequency of the periodic signal is the harmonic frequency of the piston pump, which equals the number of pistons multiplied by the rotating frequency. 

**Figure 6:**
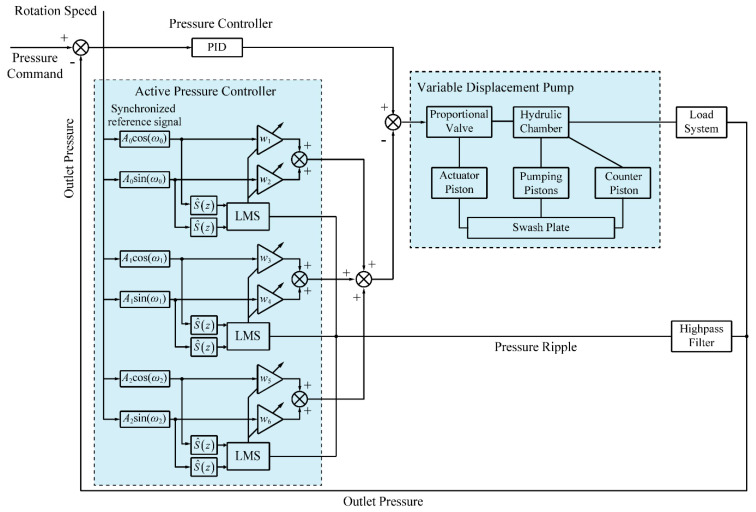
Block diagram of active pressure ripple control system.

Hence, the active pressure ripple control system includes a feedback controller and a feedback feedforward controller. As depicted in Figure 6, a feedback controller is designed to achieve a close loop pressure control with a PID algorithm. The feedforward controller uses an adaptive notch filter as an active pressure controller to reduce the pressure ripple. The frequency of the synchronized reference signal is obtained according to the rotational speed measured tachometer and the first- to third-order harmonics are taken into consideration.

There is also the fact that the summing junction in Figure 6 is different from that in Figure 5 while applying the adaptive filter to APC. There is a so-called “secondary-path” from the control valve to the pressure transducer. A direct influence is that a time delay can be introduced into APC system if the secondary-path transfer function *S*(*z*) is not compensated. Therefore, it is necessary to compensate for the secondary-path transfer function which includes the digital-to-analog (D/A) converter, proportional valve, swash plate control system, pressure transducer, and analog-to-digital (A/D) converter. A most effective method is using the filtered-x least mean square (FxLMS) algorithms to get a secondary-path estimation $\hat{S}\left(z\right)$ [30,31,32]. In this research, the secondary path is taken as a pure delay and the compensator can be modeled as $\hat{S}\left(z\right)=z^{-\Delta }$.

### 3.2. Simulation Results

**Figure 7:**
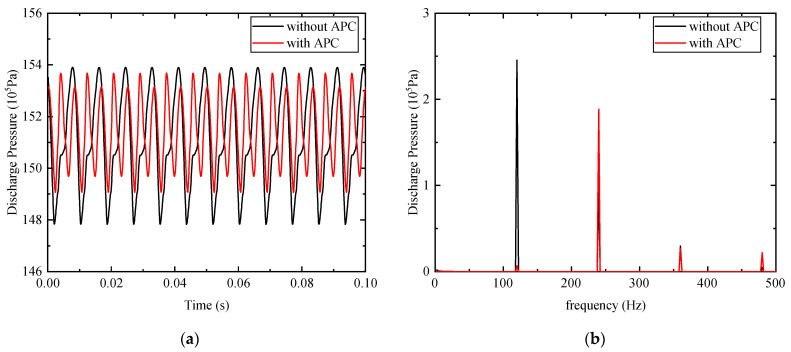
Comparisons of the discharge pressure with and without APC for the first-order frequency component: (**a**) time-domain discharge pressure; (**b**) FFT of pressure ripple.

Simulation research has been conducted to validate the possibility of the proposed method. As shown in Figure 4, the component of the first-order frequency contributes most to the pressure ripple. Therefore, the reference input was first regarded as a single frequency component. Figure 7a compared the discharge pressure ripple with and without APC for the first-order component. The peak to peak value of the discharge pressure decreases from 0.612 to 0.461 MPa. The suppression effect of 24.6% is achieved under the working condition of 800 rpm rotational speed, 12° swash plate angle, and 15 MPa outlet pressure, and the FFT of the pressure ripple is calculated and shown in Figure 7b. The graph demonstrates that the amplitude of the pressure ripple is reduced from 0.245 to 0.00675 MPa at the fundamental frequency. The reduction of pressure ripple at the first-order frequency component is remarkable, however, an increase in the amplitude of the second-order harmonic frequency can be observed. The amplitude increases from 0.0995 to 0.188 MPa, which manifests there are two peaks in one pressure signal periodic. This can be interpreted as the fact that only the first three harmonics are taken as an input of the LMS algorithm.

**Figure 8:**
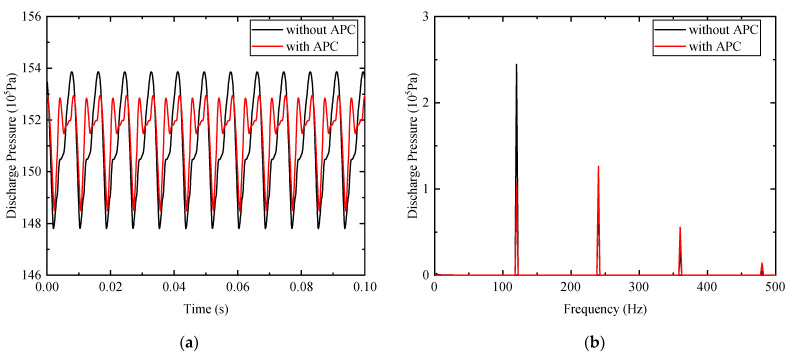
Comparisons of the discharge pressure with and without APC for multi-frequency components: (**a**) time-domain discharge pressure; (**b**) FFT of pressure ripple.

Another simulated result about the APC for the multi-frequency component is presented in Figure 8. The peak-to-peak value of the pressure ripple with APC is 0.447 MPa. Better performance of the pressure ripple reduction, about 27.0%, is obtained. From the FFT spectrum shown in Figure 8b, the ripple amplitudes of the first- to third-order frequency components with APC are 0.107, 0.126, and 0.0555 MPa, respectively. Correspondingly, the amplitudes without APC are 0.245, 0.0994, and 0.0296 MPa. The suppression effect of the first-order frequency component is about 56.3%, which is inferior to the result depicted in Figure 7, and the second-order harmonic is smaller than that for the signal-frequency APC.

## 4. Analysis and Discussion

So far, this paper has focused on the effectiveness of APC. In order to develop the active controller in future work for better performance, however, the principle of APC should be figured out. In general, the pressure ripple is chiefly determined by the discharge flow fluctuation and downstream impedance of the pipeline. As the swash plate control system is supplied internally, the load flow is a combination of the flow from pumping pistons and control system besides compression flow. The relationship can be explained as:

(24) $$Q_{\mathrm{load}}=Q_{p}+Q_{c}+Q_{\mathrm{cp}}-\frac{V_{0}}{K_{e}}\frac{dp_{s}}{dt}$$

where *Q*_p_ is the flow rate from nine pistons to discharge port, *Q*_c_ is the flow rate from actuator piston chamber to discharge port, *V*_0_ is the volume of the discharge chamber.

As mentioned in the above section, the additional control signal produced by the active pressure controller is made up of sinusoidal signals with different frequencies. The major question is how the sinusoidal signal affects the discharge flow rate to the load, as the pressure ripple is associated with flow fluctuation. Thus, the simulated results in which the multi-frequency reference input has been used for APC are analyzed.

**Figure 9:**
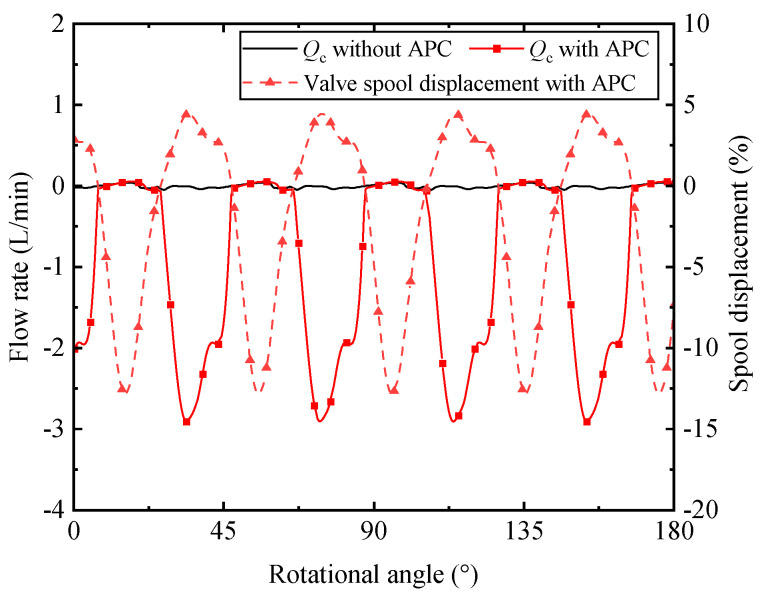
The effect on the flow from actuator piston chamber and spool displacement.

At first, the spool displacement of the control valve varies rapidly due to the sinusoidal signal with harmonic frequency, which can be shown in Figure 9. The red dashed line with the triangle mark represents the spool displacement, which presents a periodic movement. It results in the flow rate entering in and leaving out of the actuator piston chamber. As the valve port P is directly connected to the discharge port of the piston pump, the load flow is directly influenced by the extra control flow. The black solid line and red solid line with the square mark represent the flow rate from the actuator piston without APC and with APC. It should be mentioned that the flow between the actuator piston chamber and the pump housing is not taken into account in this figure. The flow rate from the actuator piston without APC is rather small while the flow rate with APC cannot be neglected. Compared with spool displacement, the reverse waveform can be seen in the flow curve with APC. The negative value of *Q*_c_ means that the flow rate drains into the actuator piston chamber while the spool displacement is positive. The negative value appears around the rotational angle of 21.3° to 50.1° and reaches a peak value of −2.91 L/min at 33.8° rotational angle. The flow rate is originated from the movement of the valve spool, which requires the corresponding dynamic response of the valve.

**Figure 10:**
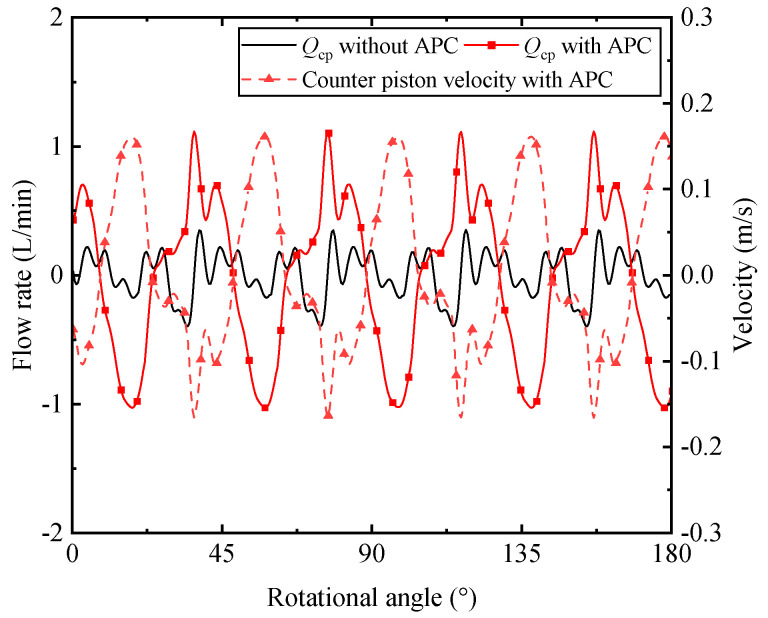
The effect on the flow from counter piston chamber and counter piston velocity.

Once the control flow alters, the movement of the swash plate is changed. As another part of control mechanism, the counter piston executes reciprocal movement along with the swash plate oscillation. This can be observed by the velocity of counter piston in a red dashed line with triangle mark as shown in Figure 10. The red solid line with square mark represents the flow rate from the counter piston with APC, which shows the opposite variation of the velocity. The positive value of the velocity means the increasing swash plate angle. Once the swash plate angle increases, the flow rate flows from the discharge port to the counter piston chamber, and vice versa. Additionally, the small flow rate without APC can be seen from this figure. The flow rate is likely to be related to the amplitude of the swash plate oscillation according to Equation (16).

The above analysis is concerned about the effect of active pressure controller on the control mechanism. The active pressure controller produces an additional control signal, which results in a regular flow rate from the control mechanism.

**Figure 11:**
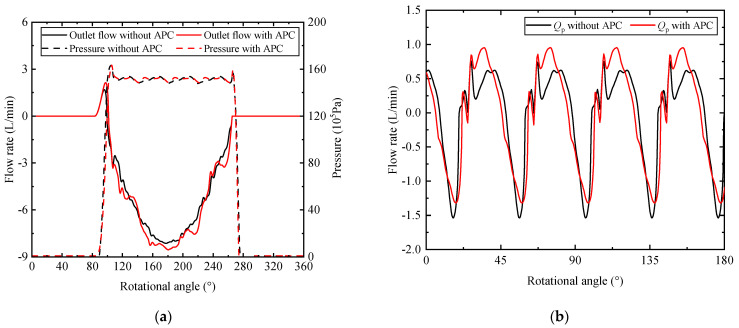
The effect on pumping pistons: (**a**) flow and pressure of a single piston; (**b**) sum of the flow.

What is more, the swash plate oscillation has been proved to do affect the pumping dynamic in recent studies [33]. To be specific, the additional movement of the piston, caused by the swash plate oscillation, leads to the variation of the flow in and out of the piston chamber. Figure 11a presents the outlet flow rate and the pressure of a single piston. The discrepancy of the small flow ripple can be seen from the solid line. This velocity of the piston due to the swash plate oscillation can explain this. As a result, the outlet flow of nine pistons is shown in Figure 11b, and the mean value of the outlet flow rate is subtracted from the instantaneous value. The peak-to-peak values are 2.30 L/min without APC and 2.27 L/min with APC. Although the variation of the flow is small in terms of quantity, the discrepancy of the wave shape can be observed. Compared with the minimum value of −1.54 L/min around the rotational angle of 16.0°, the minimum value with APC is −1.32 L/min around the rotational angle of 18.0°. A smaller flow ripple and an angle delay are found with APC. Concerning the maximum value, a crest appears around the rotation angle of 34.8° which is between the two peaks of the flow curve without APC. It is worth mentioning that the pressure transition in the piston chamber has been changed with APC as depicted in Figure 11a. It leads to the variation of the piston forces acting on the swash plate. As these forces are the excitation forces of axial piston pump vibration, it is promising to apply the methodology to the active noise and vibration control of axial piston pump.

From the above analysis concerning pumping pistons, the peak value and angular position of the flow rate from nine pistons have been altered owing to the swash plate oscillation caused by APC. Additionally, the changing piston dynamics could be utilized to reduce the structure-borne noise.

**Figure 12:**
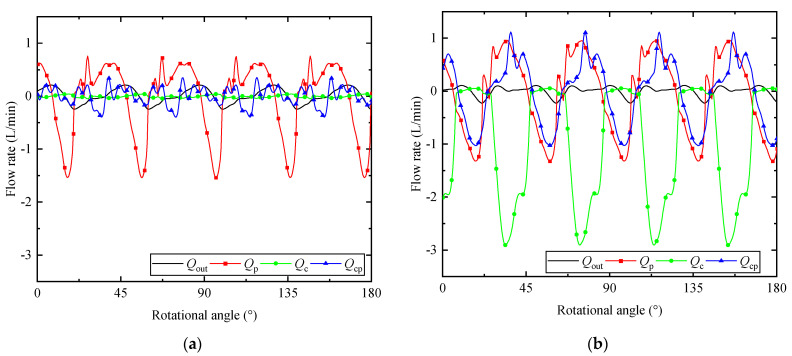
Flow rates from different chambers: (**a**) without APC; (**b**) with APC.

On the whole, it is possible for us to figure out the reason for the reduction of pressure ripple. Figure 12a summarizes the flow rates from different parts to the load without and with APC. The red, green, and blue lines represent the flow rates from the nine pistons, the actuator piston chamber, and the counter piston chamber, respectively. The black line is the flow rate flow into the system load. It can be seen that the largest fluctuation without APC is the flow from the nine pistons, which demonstrates the flow fluctuation is primarily decided by the flow from the nine pistons. As a result, the load pressure fluctuates with the outlet flow fluctuation. 

Considering the scenario when the APC is applied, the results of the flow rates are shown in Figure 12b. The major difference between flow rates with and without APC is the flow of the actuator piston caused by the spool displacement. Obviously, a trough appears in the flow curve of the actuator piston with APC, which is opposite to the outlet flow of nine pistons. Negative flow from actuator piston and positive value of flow from nine pistons are in a similar range of rotational angle. It balances out the flow fluctuation caused by the nine pumping pistons to some extent. Additionally, the flow fluctuation shape of the flow rate from nine piston chambers is changed in comparison to the results without APC. This can be attributed to the swash plate oscillation. Besides, the oscillation of the swash plate gives rise to the flow from the counter piston. Taking the rotational position of peak value in the flow curve into consideration, the flow from counter piston performs a negative effect on the pressure ripple reduction. Fortunately, the outlet flow is not the simple addition of different flows. For instance, the minimum value of the outlet flow *Q*_out_ is not equal to the sum of the minimum value of *Q*_p_ and *Q*_cp_.

The pressure ripple reduction can be achieved with the active pressure controller. Compared with the manual adjustment from Ohuchi et al. [21], the active pressure controller is capable of calculating the optimal amplitude and phase of the additional sinusoidal signal. This makes it better suited to the varying working conditions of variable displacement pumps. The proposed approach mainly makes use of the effect of the variable mechanism on the pressure ripple instead of the swash plate oscillation, which means that the modification of the control system such as that in Casoli et al. [25] is not required to achieve the large oscillation of the swash plate.

## 5. Conclusions

In this paper, an active pressure pulsation control method has been investigated in a self-supplied variable displacement pump. Taking the dynamics of the pumping pistons and control system into consideration, a simulation model is established to calculate the pressure pulsation. Experimental research has been carried out and the established model has been validated by the discharge pressure. An active pressure controller utilizing the filtered-x least mean square algorithm with delay compensation is designed for pressure ripple reduction. The active pressure control for the single-frequency and multi-frequency components has been studied and the results show the effectiveness of the proposed methodology for pressure ripple reduction. About 24.6% and 27.0% reduction of the amplitude can be realized by a single-frequency algorithm and multi-frequency algorithm, respectively. 

In addition, as the swash plate control system is supplied internally, the reduction of the pressure pulsation should be attributed to the comprehensive reaction of different flow rates. Thus, the theoretical analysis has been conducted to reveal the principle of active pressure control and summarized as follows:

1. The amplitude and angular position of the peak value in the flow curve have been changed due to the swash plate oscillation and valve spool movement. The major reason is that the flow from the actuator piston neutralizes the peak value of the flow ripple generated by the nine pistons and the counter piston. The pumping dynamic has been altered due to the larger swash plate oscillation, which might account for the vibration and noise reduction.
2. For a better suppression effect, the higher frequency response of the control valve is required. In general, the amplitude of the swash plate oscillation is limited by the poor dynamic characteristic of the swash plate control system, which restricts the utility of the nine pistons flow.
3. Like other active pressure control techniques, the proposed method affected the volumetric efficiency due to the additional flow to the tank port, and the reciprocal motion of the control valve does challenge its reliability. In future work, a possible approach to tackle this issue could be to combine the passive technique in the high frequency with the active technique in the low frequency.

## Data Availability

Not applicable.
